# Rare Earth Elements Recovery Using Selective Membranes via Extraction and Rejection

**DOI:** 10.3390/membranes12010080

**Published:** 2022-01-11

**Authors:** Atiyeh Bashiri, Arash Nikzad, Reza Maleki, Mohsen Asadnia, Amir Razmjou

**Affiliations:** 1 School of Metallurgy and Materials Engineering, Iran University of Science and Technology, Tehran 16845-161, Iran; atie_bashiri@metaleng.iust.ac.ir; 2 Department of Mechanical Engineering, University of British Columbia, 2054-6250 Applied Science Lane, Vancouver, BC V6T1Z4, Canada; arash.nikzad@mech.ubc.ca; 3 Department of Physics, University of Tehran, Tehran 14395-547, Iran; rezamaleki96@gmail.com; 4 School of Engineering, Macquarie University, Sydney, NSW 2109, Australia; mohsen.asadnia@mq.edu.au; 5 UNESCO Centre for Membrane Science and Technology, School of Chemical Engineering, University of New South Wales, Sydney, NSW 2052, Australia

**Keywords:** rare earth elements (REEs), polymer inclusion membrane (PIM), ion-imprinted membrane (IIM), nanocomposite membrane, solid membrane

## Abstract

Recently, demands for raw materials like rare earth elements (REEs) have increased considerably due to their high potential applications in modern industry. Additionally, REEs’ similar chemical and physical properties caused their separation to be difficult. Numerous strategies for REEs separation such as precipitation, adsorption and solvent extraction have been applied. However, these strategies have various disadvantages such as low selectivity and purity of desired elements, high cost, vast consumption of chemicals and creation of many pollutions due to remaining large amounts of acidic and alkaline wastes. Membrane separation technology (MST), as an environmentally friendly approach, has recently attracted much attention for the extraction of REEs. The separation of REEs by membranes usually occurs through three mechanisms: (1) complexation of REE ions with extractant that is embedded in the membrane matrix, (2) adsorption of REE ions on the surface created-active sites on the membrane and (3) the rejection of REE ions or REEs complex with organic materials from the membrane. In this review, we investigated the effect of these mechanisms on the selectivity and efficiency of the membrane separation process. Finally, potential directions for future studies were recommended at the end of the review.

## 1. Introduction

**Figure 1:**
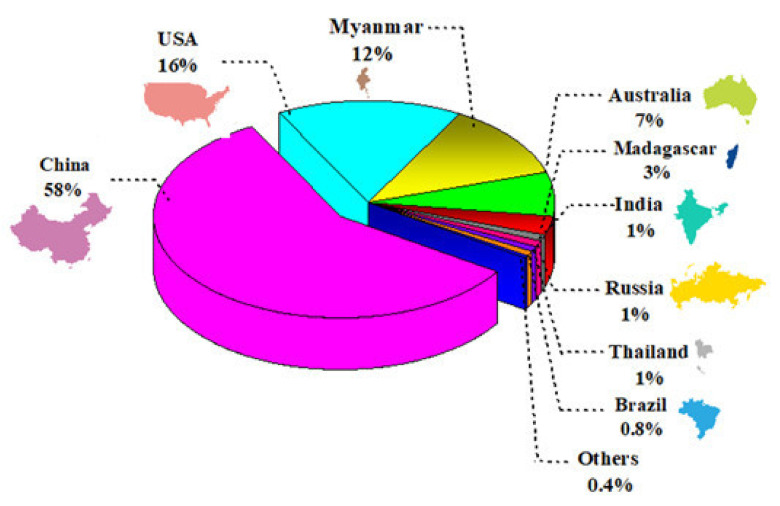
Schematic diagram of production shares of REEs from different countries around the world in 2020, illustrating that China has the highest production share of up to 58%, followed by USA, Myanmar, and Australia, as mentioned on the diagram [14].

In recent years, the consumption of scarce raw materials such as rare earth elements (REEs) has exponentially increased due to their fundamental requirements in advanced technologies and industrial applications [1]. Lots of cutting-edge techniques such as new catalysts in petroleum refining, rechargeable batteries for electric vehicles, phosphors in flat panel display and ceramics presently need the REEs-based materials [2,3,4,5,6]. Owing to the necessity of REEs, they are famous as “the vitamins of modern industry” [7]. REEs include a large metallic group of 17 SC and Y elements besides the 15 lanthanide elements, La, Pr, Ce, Pm, Nd, Sm, Eu, Gd, Dy, Tb, Ho, Tm, Er and Yb [8,9], which typically consist of light REEs (LREEs, La-Eu) and heavy REEs (HREEs, Y and Gd-Lu) categories. REEs generally have similar electronic configurations, resulting in similar chemical and physical properties [10,11]. The REEs are not rare compared to precious metals, but their low concentrations (about ppb) and vast locations make them challenging to collect [2,8]. The main ores of REEs comprise monazite, xenotime, lanthanite, allanite, bastnäsite, loparite and phosphate rocks [3]. Currently, China is the leading REEs producer, accounting for about 90% of REEs worldwide [12]. Nevertheless, China has limited its REE exports due to growing domestic demand, leading to a global REE supply shortage [2,13]. Figure 1 shows the diagram of production shares of REEs from different countries around the world in 2020 [14].

Ironically, the industrial separation from REEs ores involves severe environmental pollution and an increased economic price of these elements. In addition, because REEs have identical properties, their separation is a complex process [15]. Therefore, the REEs recovery from wastewaters is of great importance and reduces their production cost [4,16]. Meanwhile, recovering REEs from industrial wastewaters as secondary resources is still a major challenge due to low concentrations. So far, various technologies have been applied to remove and recover REEs from different contaminated wastewaters, such as solvent extraction [17], ion exchange [18], precipitation [19,20], adsorption [21,22], and electrochemical processes [23]. Because of its large production capacity and fast mass transfer, the primary industrial-scale method is a solvent extraction from their leaching solution [24]. However, this is still facing some challenges such as low selectivity and purity of the extracted elements, remaining large amounts of wastes and high consumption of reagents [25]. In the precipitation process, created sludge needs further treatment. In addition, the attendance of other ions in the obtained stream can affect the extraction efficiency [26]. Also, a purification step after adsorption is required for the adsorption process, which is associated with consuming chemical materials and increasing costs [27,28]. Thus, new separation methods need to be developed for the efficient extraction of REEs from secondary resources. Recently, membrane separation technology (MST) has attracted a lot of attention due to its porous structure, good selectivity and environmentally friendly essence. In this study, we evaluate MST for the recovery of REEs. The review provides the REEs separation mechanisms from various membrane techniques. The structure, selectivity and feasibility of these membranes are also investigated.

## 2. Membrane Separation Technology (MST) for Recovery of REEs

MST, a combined process of simultaneous removal and extraction without the need for thermal operations, can be a promising method for a green separation approach. In recent years, the use of membrane technology for the recovery of REEs from effluent and wastewater due to many advantages, such as high selectivity and recovery, easy operation, the minimum created volume of sludge and the limited production of waste material, has attracted much attention [29]. Significant research on the membrane process for REEs separation has involved the liquid-liquid extraction method. Although this method is the most used and conventional way to extract REEs on an industrial scale [30], consistently low extraction efficiency, the presence of impurities in the final product, a low contact area and loss of the extractant in the aqueous phase are still present in large-scale processes [31]. To solve these problems, various types of liquid membranes (LMs), such as emulsion liquid membrane (ELM), bulk liquid membrane (BLM) and electrostatic quasi-liquid membrane (ESPLM), have been developed to improve the extraction of REEs [32,33,34,35,36,37]. In all these membranes, the separation mechanism is generally permeating lanthanide ions (Ln^3+^) from feed solution through an organic phase to the stripping phase due to the concentration gradient. However, because of the weak stability and low membrane surface of these methods, which is specified by the different solubility of the phase, new methods such as the supported liquid membrane (SLM) [38,39,40,41,42] and the hollow fiber supported liquid membrane (HFSLM) [43,44,45,46,47,48]—upon which extractant is embedded on the porous support—have been developed [16]. For example, J. Martínez et al. [49] applied an effective flat sheet-supported liquid membrane (SLM) for the recovery of Y^3+^-Nd^3+^-Dy^3+^, using bis (2-ethylhexyl) hydrogen phosphate (D2EHPA) as an extractant and polymeric support. However, the most significant disadvantage of supported liquid membranes is that an aqueous solution usually wets the porous structure support and is inevitably blocked by carriers. A solvent extraction process followed by a hollow fiber-supported liquid membrane (HFSLM), with a 1 mol/L D2EHPA extractant, was studied by Achmad et al. [17]. The best recovery percentage achieved at 90 min for Nd^3+^, Dy^3+^ and Pr^3+^ was 63.13%, 15.21% and 56.29%, respectively.

Although the application and development of these methods have shown significant separation effects, their utilization on an industrial scale still involves some problems. As a result, the REEs separation processes have shifted towards applying stable non-liquid membrane processes, in which carriers are chemically or physically attached to the membrane or a porous supporting structure [50]. Therefore, they can be used without the concern of extractant loss into the aqueous phase. Polymer inclusion membrane (PIM) is a relatively new method for the solid extraction of REEs, which is inexpensive and has high flux and mechanical flexibility [51,52,53] 

Other solid extraction methods for separating REEs are mainly included in ion-imprinted techniques [54,55,56,57] on polymer membranes. Some other membranes, such as metal-organic frameworks (MOFs) membranes, carbon membranes and stainless-steel membranes, due to their unique properties, also have a high potential for separation of REEs [58,59,60,61]. The separation mechanism of these methods is mainly involved in the adsorption or rejection of target ions from the surface and the transition of other ions through the membrane. Consequently, the extraction and separation mechanism of REEs can be categorized into three groups:

1. Diffusion/permeation mechanism.
2. Facilitated/retarded permeation mechanism.
3. Rejection mechanism.

In the following sections, the operation of these mechanisms for the extraction of REEs is discussed in detail. Also, the advantages and disadvantages of separating different REEs from various waste streams with respect to the use of these mechanisms, have been investigated.

## 3. Diffusion/Permeation Mechanism

**Figure 2:**
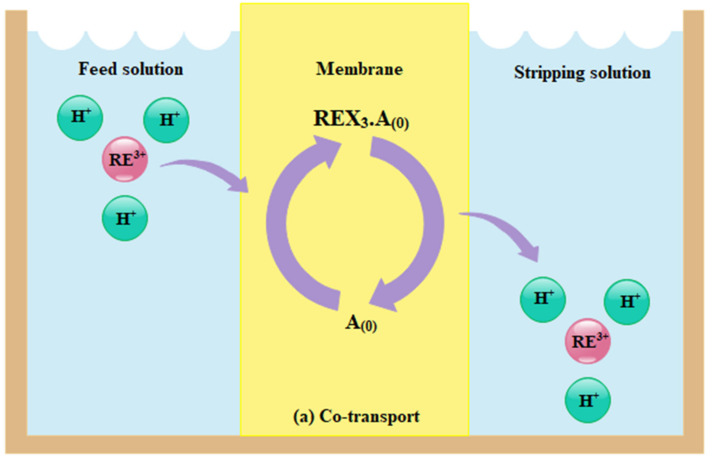

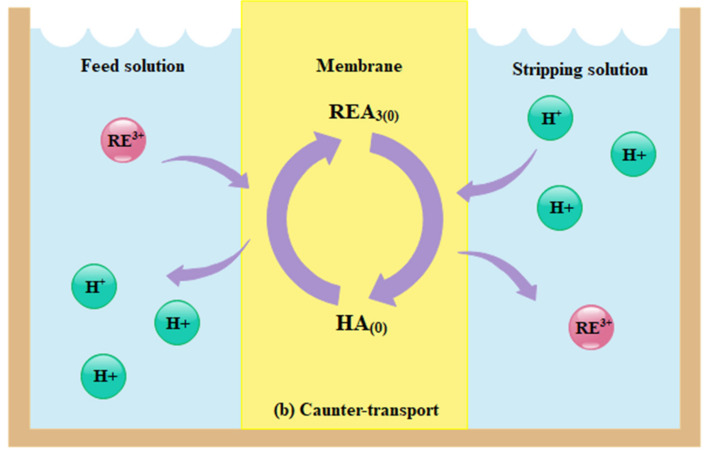
The extraction mechanism of REEs through permeable membranes, (**a**) cotransport of both REE ions and anionic specious by mainly neutral organic extractant and (**b**) counter-transport of REE ions and H^+^ due to concentration differences of H^+^ between both sides of membrane by mainly acidic organic extractant [4].

As illustrated in Figure 2, the extraction of REE ions in a permeable membrane occurs through co-transport and counter-transport mechanisms. In the co-transport mechanism, the RE^3+^ and its anions such as NO_3_^−^, Cl^−^ or SO_4_^2−^ make complex with organic extractant (according to Equation (1)) and diffuse together through the membrane, appropriate for neutral extractants.

(1) $${\mathrm{RE}}^{3+}+3X^{-}+{\;A}_{\left(0\right)}↔{\;\mathrm{REX}}_{3}.A_{\left(0\right)}\;$$

In the counter-transport mechanism, the RE^3+^ makes a complex with organic extractant a (according to Equation (2)) and diffuses through the membrane due to cation exchange with H^+^, which usually happens for acidic extractants. Therefore, the permeation of REE ions depends on the gradient of H^+^ concentration from feed solution to stripping solution. In these membranes, the extraction is influenced by the structure of support or membrane matrix, the kind of extractant, the presence of competitive ions, the PH of the feed solution and stripping solution and the concentration of initial concertation [4].

(2) $${\mathrm{RE}}^{3+}+3{\mathrm{HA}}_{\left(0\right)}↔{\;\mathrm{REA}}_{3\left(0\right)}+3H^{+}\;$$

It is worth noting that the permeation coefficient is a key parameter for these mechanisms to evaluate the efficiency of extraction of REEs and is calculated according to Equation (3):

(3) $$Ln\left(\frac{{\left[{\mathrm{RE}}^{3+}\right]}_{feed,t}}{{\left[{\mathrm{RE}}^{3+}\right]}_{feed,0}}\right)=-\frac{APt}{V}$$

where [RE^3+^]*_feed,t_*, [RE^3+^]*_feed_*_,0_, *A*, *V*, *t* and *P* are the REE ions concentration in the feed solution at *t* time, initial REE ions concentration in the feed solution, the membrane active surface area, the volume of source solution, the time and the permeability coefficient, respectively.

### 3.1. Supported Liquid Membranes (SLMs)

The liquid membrane technique (LMT) is a huge category of extraction processes, in which transportation of extracted specious occurs through the membrane phase. Liquid membranes are classified into unsupported membranes such as emulsion liquid membranes (ELMs), bulk liquid membranes (BLMs) and supported liquid membranes (SLMs). SLMs constructed of a porous support and carrier to extract target ions have become an effective system due to their high selectivity and lower carrier consumption. SLMs have been widely used for the extraction of REEs. However, the most significant disadvantages of SLMs are low stability and block support pores by aqueous solutions. So, new approaches have been applied for a more effective separation of REEs by SLMs. Liang et al. [39] studied a novel hybrid flat renewal SLM with a dispersion phase using D2EHPA as the extractant and HNO_3_ as the stripping phase for Nd^3+^ extraction. The dispersion phase was composed of the membrane phase (carriers dissolved in organic solutions) and the stripping phase. The stripping phase concentrated the REE ions after suspending and still standing in the dispersion phase. The dispersion stripping phase also preserved a renewable supply for the extractant, which would prevent the re-impregnation of typical SLM and stabilize the membrane phase fixed in the matrix phase. In an optimum condition such as 0.1 mol/L D2EHPA, 4 mol/L HNO_3_, 2 × 10^−4^ mol/L, an initial concentration of Nd^3+^ and feed solution PH pf 4.6 and a time of 75 min, the extraction of Nd^3+^ was near 92.9%. The mechanism of REEs extraction from the SLM process using D2EHPA as an extractant from coal fly ash leachate is reported by Smith et al. [22].

Cui et al. [62] investigated the transportation mechanism of an SLM with PVDF support and TODGA carrier. PVDF membrane was selected as the support due to its high thermal, chemical and mechanical stability. The extraction of REEs with TODGA in HNO_3_ media follows Equation (4):

(4) $${\mathrm{RE}}^{3+}+3{\mathrm{NO}}_{3}^{-}{}^{\;}+3{\mathrm{TODGA}}_{\left(0\right)}↔{\left(\mathrm{TODGA}\right)}_{3}.\mathrm{RE}{\left({\mathrm{NO}}_{3}\right)}_{3\left(0\right)}$$

the sign (0) refers to the organic phase, so the extraction constant of RE^3+^ would be calculated according to the Equation (5):

(5) $$K_{H^{+}}=\frac{\left[{\left(\mathrm{TODGA}\right)}_{3}.\mathrm{RE}{\left({\mathrm{NO}}_{3}\right)}_{3}\right]}{\left[{\mathrm{RE}}^{3+}\right].{\left[3{\mathrm{NO}}_{3}^{-}{}^{\;}\right]}^{3}.{\left[\mathrm{TODGA}\right]}^{3}}$$

**Figure 3:**
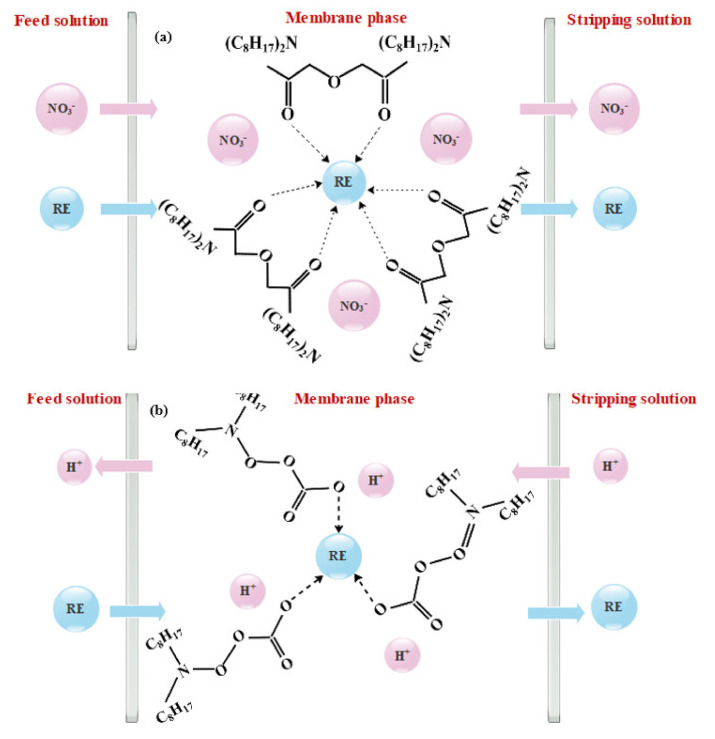
(**a**) the schematic of REE ions extraction through the supported liquid membrane, both REE ions and NO_3_^−^ pass through the membrane by formation complex with TODGA carriers, which are a neutral extractant, by co-transport mechanism [62]; (**b**) the schematic of REE ions extraction through the supported liquid membrane, REE ions pass through the membrane by formation complex with DODGAA carriers, which are acidic extractants by counter-transport mechanism [63].

The REEs extraction constants reported as K_La_^3+^ = 5.08 × 10^3^, K_Pr_^3+^ = 9.40 × 10^3^, K_Ce_^3+^ = 1.28 × 10^4^, K_Nd_^3+^ = 7.71 × 10^3^. The increase in acidity caused the promotion of the complex formation. Nevertheless, with the acidity increase, nitric acid was also extracted, which led to the occupation of extractants by the HNO_3_ molecules and decreased extraction efficiency. So, they used a mixture of HNO_3_ and NaNO_3_. By increasing NO_3_^−^ ions from 2 mol/L to 3 mol/L, the best Nd^3+^ transportation of 99% was achieved. The transportation mechanism in the membrane phase is depicted in Figure 3a. The REE ion bound to the extractant, and a charge balance was reached with the formation of the neutral complex with NO_3_^−^. The complex then transfers from feed solution to membrane phase because of higher distribution, and thus REEs are released into the stripping solution.

Asadollahzadeh et al. [64] studied a synergistic extractant containing [C_6_MIM][NTf_2_] ionic liquid, trioctyl phosphine oxide (TOPO) and Tributyl phosphate (TPB) for the extraction of Nd^3+^ from Pr^3+^ using a supported liquid membrane system. The results show that when the TOPO was used as an extractant in the 1.8 M H_2_SO_4_ stripping phase, the permeation coefficient for Nd^3+^ and Pr^3+^ were 0.8627 × 10^6^ m/s and 0.2418 × 10^6^ m/s, respectively, and when TPB was used, the permeation coefficient for Nd^3+^ and Pr^3+^ was 0.4043 × 10^6^ m/s and 0.0910 × 10^6^ m/s, which indicate that the separation of Nd^3+^ from Pr^3+^ was not acceptable for both extractants. However, in the case of using both TOPO and TPB, the permeation coefficient for Nd^3+^ and Pr^3+^ changed to 1.4059 × 10^6^ m/s and 0.6275 × 10^6^ m/s, respectively. However, when [C_6_MIM][NTf_2_] ionic liquid was added to TOPO and TPB, the obtained permeation coefficients for Nd^3+^ of 2.3200 × 10^6^ m/s and Pr^3+^ of 0.7112 × 10^6^ m/s, had considerable differences. Consequently, the utilization of ionic liquids can be a beneficial approach for enhancing the selectivity of SLMs. Baba et.al [63] used [C_8_mim][Tf2N] as a green solvent and DODGAA as the extractant for the separation of Dy^3+^ and Nd^3+^ against ferric ions by the SLM method. After 30 h, up to 100% of REE ions were transported with just 10% transportation of ferric ions. The extraction constant of Dy^3+^ and Nd^3+^ were reported as 6.26 × 10^−1^ and 2.35 × 10^−2^, respectively, which shows the separation capability of Dy^3+^ and Nd^3+^ after the separation of Fe ions by this membrane. The transportation mechanism in the membrane phase is illustrated in Figure 3b. In Table 1, the information about some SLMs used for REEs extraction is summarized.

As discussed, the SLMs have good potential for utilization in REEs extraction. Despite the BLMs and ELMs, they use lower chemical materials and are more easily operable with more efficiency. So, they can be applied in industrial applications; however, some problems such as weak stability due to pore blockage and low lifetime still need to be eliminated.

### 3.2. Polymer Inclusion Membranes (PIMs)

**Figure 4:**
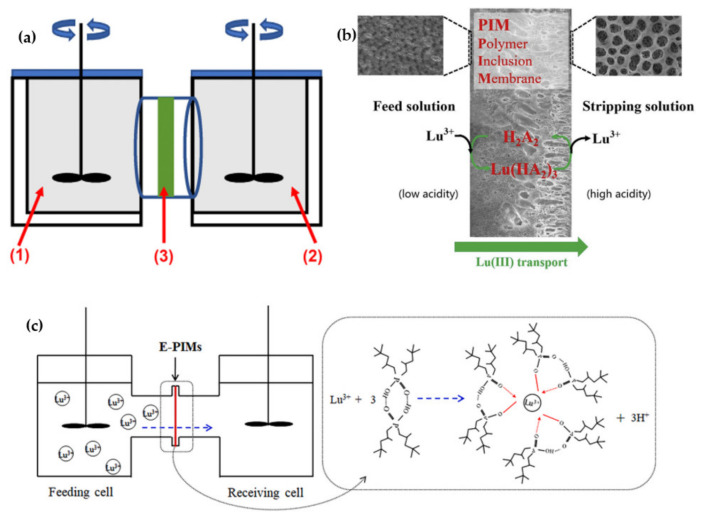
(**a**) Transport apparatus for extraction of Lu^3+^ by a P227@PVDF by simultaneously extracting and recovering Lu^3+^ in one device ((1) feed solution, (2) stripping solution and (3) polymer inclusion membrane), adapted from Ref. [7]. Copyright 2021 Elsevier; (**b**) extraction and stripping of Lu^3+^ by P227@PIM, adapted from Ref. [69]. Copyright 2020 Elsevier; the surface morphology of membrane indicate the asymmetric structure of membrane with hierarchically ordered pores. The pore sizes in the inner layer and outer layer are ~1 μm and ~8 μm, so the P277@ PIM (40 wt%) have high accessibility to the solution causing the increase of transportation of Lu^3+^; and (**c**) cation exchange transport mechanism of Cyanex272 and Lu^3+^ ion by E-PIMs, which shows the Lu^3+^ forms complex with a functional group (−OH) of extractant and released the H^+^, adapted from Ref. [70]. Copyright 2021 Elsevier.

Polymer inclusion membranes (PIMs), as a new class of polymeric membranes, have recently attracted more attention for the extraction of metal ions, especially REEs [52,67]. In PIM membranes, the extraction process with stripping procedure is combined simultaneously into one device. Moreover, due to long-time stability, low carrier loss, elimination of large volumes of diluents and no problems with phase separation, it seems that PIM could be a potential green alternative technology for concentrating, separating and recovering REEs [4,68]. Figure 4a draws a typical diagram of the transport apparatus for the extraction of Lu^3+^ by a P227@PVDF polymer inclusion membrane [7]. Thus, the separation process becomes more straightforward and occurs continuously. Despite the benefits of PIM, very little research has been carried out on the extraction and recovery of REEs to date.

Huang et al. [7] used a new PIM for Lu^3+^ separation and extraction from a solution that contained Lu^3+^, La^3+^ and Sm^3+^. The membrane composition was PVDF with 60 wt% as the primary matrix and 40 wt% P227 (di(2-ethylhexyl) phosphinic acid) as the extractant and the plasticizer. A structure with hierarchically ordered pores of P227@PIM (40 wt%) due to high surface roughness and a large contact area with the solution exhibited improved membrane accessibility to the solution. Moreover, the membrane acted as an effective selective transporter of Lu^3+^ from the small pore side to the large pore side. As the P227 is an acidic extractant, the PH has a crucial role in the membrane’s selectivity. At PH = 1.5 after five h, the recovery factor of Lu^3+^, La^3+^ and Sm^3+^ were 85%, 40% and 4%, respectively. After the increase of PH to 2.4, the Sm^3+^ was selectively separated from La^3+^. However, with a further increase of PH, the La^3+^ separation reached 50%. This indicated that P227@PIM (40 wt%) could be an excellent membrane for separating heavy and light REEs from each other. Carrier content, stirring speed, temperature and stripping acidity also affected the PIM transport efficiency. They reported that the mechanism of extraction and stripping Lu^3+^ by P227 existed in the dimer form in PIM, which is a cation exchange process. Briefly, Lu^3+^ and the extractant carrier first contact the interface between the membrane and the feed solution to form a complex. In the second stage, the complex diffuses through the membrane into the stripping solution due to differences in both sides’ acidity and the gradient concentration of Lu^3+^ and H^+^ as driving forces. In the end, Lu^3+^ from the extracted complex is replaced by H^+^ at the membrane interface and the stripping solution and is released into the stripping solution. The process schematic is shown in Figure 4b. The transport efficiency of Lu^3+^ after 5 h and 12 h reached 85% and 96%, respectively [69].

Chen et al. [70] have also reported the synthesis of a novel modified EVOH polymer inclusion membrane with Cyanex272 as a carrier to separate Y^3+^ and Lu^3+^. They concluded that the utilization of EVOH caused no difference between P = O, P − OH in Cyanex272 and Lu^3+^ coordination. SEM and AFM analyses indicated that adding a suitable amount of EVOH could produce larger surface pores and internal channels. The membrane permeability coefficients of Lu^3+^ and Yb^3+^ were 114.82 μm/s and 156 μm/s, respectively; the primary fluxes were also 65.61 μm/m^2^.s and 190.17 μm/m^2^.s, respectively. The Lu^3+^ and Yb^3+^ separation factor was measured as 1.37 in the optimized condition. All obtained results demonstrate that this novel synthesized PIM with serving EVOH as a hydrophilic additive can be further used to extract heavy REEs. Figure 4c shows a brief illustration of the cation exchange transport mechanism of Cyanex272 and Lu^3+^ in the membrane.

Kelov et al. [53] introduced a polymer inclusion membrane for the selective extraction and recovery of REE ions consisting of 45% D2EHPA and 55% PVC. The complete extraction and separation of Yb^3+^, La^3+^ and Ga^3+^ ions were attained. Selective and complete extraction of these REEs ions was reached in the different pH of the H_2_SO_4_ feed solution (Yb^3+^ at pH = 0.25, Gd^3+^ at pH = 1.25 and La^3+^ at pH = 2.25). The thermodynamic extraction constants for Yb^3+^, Gd^3+^ and La^3+^ reported as equal to 92,700, 85.5 and 0.896, respectively. The considerable difference of extraction factors caused the possibility of the separation of light, middle and heavy REE ions by varying solution acidity. Table 2. summarizes some other PIMs for the separation of various REEs. Described research demonstrates that the effective and selective transport of the desired ions by PIM depends directly on the used carrier [71]. As appropriate carriers, ionic liquids (ILs) also have reports for improving the selectivity of PIMs. Chen et al. [72] used [A336][P507] as a bifunctional ionic liquid without any plasticizer with a PVDF matrix for the extraction of Y^3+^ and Lu^3+^. PVDF matrix (62.5 wt%) and [A336][P507] carrier (37.5 wt%) had weak physical interaction and created an asymmetric structure, which accelerated the transport of Lu^3+^ from small pores in the surface with feed solution to the large pores from the surface of stripping solution. For Lu^3+^ extraction, in condition of 7.5 × 10^−4^ mol/L LuCl_3_, initial pH = 2.84, 1.0 mol/L HCl stripping solution, the permeation coefficient was 2.80 µm/s. Moreover, the Y^3+^ transportation was much faster than Lu^3+,^ more selective compared to liquid–liquid extraction. Although the ionic liquids have efficient performance on REEs extraction, some problems such as high costs and difficulty in their synthesis restricted their uses [73,74].

PIMs are generally more stable and relatively longer in the lifetime than supported liquid membranes (SLMs) and can easily be prepared. However, the main problem of these membranes is that, after a while, due to the fouling, the membrane permeability reduces. Therefore, using these membranes to extract REEs in the industry requires more improvement and development of their durability [78].

## 4. Facilitated/Retarded Permeation Mechanism

REEs family are well-known for their similar chemical and physical properties [79], such as the similarity in their radius and oxidation states (+III), which makes them hard to separate [80]. On the other hand, several REEs are valuable in economic aspects. Therefore, required methods with high-efficiency selectivity are required to extract a particular and substantial element from REEs in waste streams. Recently, the use of membranes called ion-imprinted membranes and nanocomposite membranes have been considered for the extraction of REEs due to their high selectivity to desired elements from others. In these membranes, due to the use of unique technologies in their synthesis, the target element is absorbed in places embedded on the surface or inside the membrane, and interfering ions pass freely through the membrane. Finally, the adsorbed ion can be recovered and extracted with high purity.

### 4.1. Ion Imprinted Membranes (IIMs)

**Figure 5:**
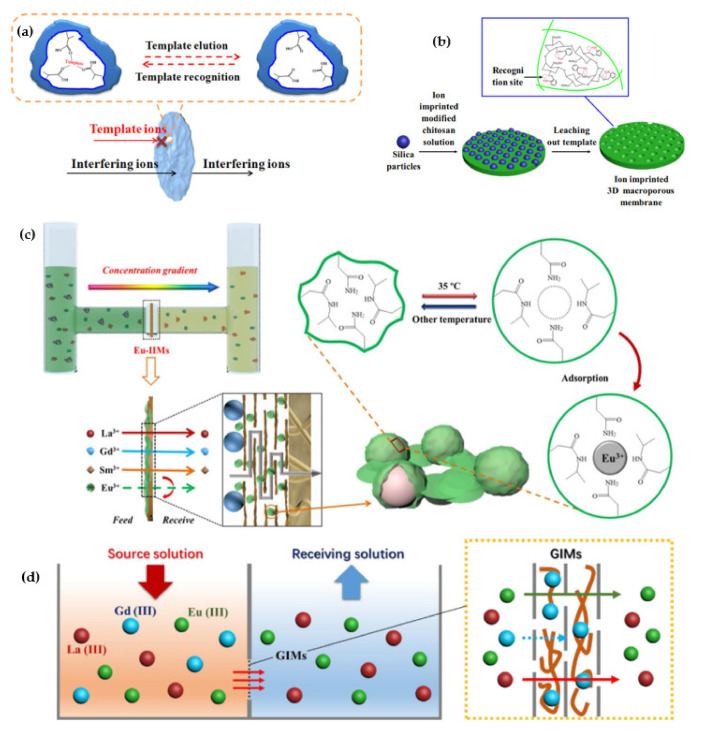
(**a**) illustration of separation mechanism of ion-imprinted membranes, which REE ions are adsorbing in ion-imprinted sites and interfering ions pass through the membrane, adapted from Ref. [74]. Copyright 2021 Elsevier; (**b**) preparation producer of Dy^3+^ ion-imprinted 3D macroporous chitosan membrane (II-MAC) via a simple immersion–precipitation–evaporation strategy, adapted from Ref. [81]. Copyright 2017 Elsevier; (**c**) producer of Eu^3+^ from Eu^3+^-imprinted membranes (Eu-IIMs) nanocomposite that shows after the creation of an active site of Eu^3+^ at 35 °C, the membrane gets stuck while the Eu^3+^ and other ions (La^3+^, Gd^3+^ and Sm^3+^) from membrane layers pass, adapted from Ref. [82]. Copyright 2018 Elsevier; and (**d**) Gd^3+^ separation schematic from a Gd^3+^-imprinted membrane (GIM) by one-dimensional carbon nanotubes and two-dimensional modified polydopamine-graphene oxide adapted from Ref. [83]. Copyright 2019 Elsevier.

The most exciting method for selectively recognizing and separating ions is the ion-imprinted technique (IIT), which has been developed extensively due to the artificial specific imprinting processes’ capability to recognize the target ions and their high tolerance to acidic and basic environments. However, owing to some problems such as poor regeneration, high diffusion barriers and difficult separation for REEs, the utilization of this method in industrial applications is still limited. The combination of the ion-imprinted technique (IIT) and membrane separation technique (MST) was proposed in past decades to solve these problems. The separation of REEs has shown high potential ion-imprinted membranes (IIMs) to absorb and separate the specific ions from REEs with extremely high selectivity. The main ways for sorption of REEs by IIMs membrane are facilitated/retarded permeation processes. As illustrated in Figure 5a, when the feed solution passes through the membrane, the template ion is adsorbed by the imprinted site, while the interfering ions pass freely through the membrane. The utilization of IIMs for the extraction of REEs were reported firstly by Mosbach et al., in 1993. Accordingly, the new synthesis method (surface imprinting) and multilevel distributed structure of IIMs on membrane surfaces have been developed for the extraction of REEs [74].

Liu et al. [81] have invented a novel Dy^3+^ ion-imprinted 3D macroporous chitosan membrane (II-MAC) by a simple immersion–precipitation–evaporation strategy for the solid–liquid selective extraction of Dy^3+^. Chitosan, a material possessing many free hydroxyl and amino agents with unshared pair electrons, can constitute complexes with REE ions. For the construction of the microporous structure, the silica particle as a porogenic agent was also incorporated in imprinted material. The effect of solution pH, initial concentration of Dy^3+^ and contact time were investigated. They showed that when the stream passed through the II-MAC membrane, the Dy^3+^ adsorbed through the rebinding of amino groups, which were positively charged during the leaching and creation of the Dy^3+^ vacancy stage Dy^3+^ ions. As shown in Figure 5b, after adsorption experiments in the optimal pH = 7 at 25 °C, the maximum Dy^3+^ adsorption capacity of the membrane Dy^3+^ was 23.3 mg/g.

Moreover, Nd^3+^, Pr^3+^ and Tb^3+^, as competitive ions with the most identical valences and radius with Dy^3+^, were selected. The Dy^3+^ distribution coefficient in the presence was 494.88 mL/g, that shows considerably higher than other ions. This novel II-MAC can improve the selective adsorption of Dy^3+^ and mass-transfer kinetics. Specifically, the prepared membrane is time-saving and convenient for Dy^3+^ extraction ions, which, after five times reusing, exhibited excellent absorption ability.

Also, Wang et al. [82] developed Eu^3+^-imprinted membranes (Eu-IIMs) nanocomposite for selectively separating Eu^3+^ from wastewaters that harm human bodies due to inhibiting prothrombin production from La^3+^, Sm^3+^ and Gd^3+^. The most important of this membrane is its anti-fouling performance due to modification of the membrane surface with Ag nanoparticles. The base of the membrane was Polydopamine (PDA) to enhance the interfacial adhesion. Graphene oxide (GO) provides a lot of oxygen-containing functional groups, such as carboxyl, hydroxyl and modified silicon dioxide (kSiO_2_), to form hydrophilic nanocomposite membranes, which improve the low liquid fluxes and were stacked on PDA. They reported that the maximum rebinding capacity could be reached at pH = 7 with a recovery percentage of 76.87%. At lower PH, due to the protonation of active sites, the adsorption capacity was decreased. Moreover, high Eu^3+^-rebinding capacity (101.14 mg/g) with adsorptive selectivity 1.82 for Eu^3+^/La^3+^, 1.57 for Eu^3+^/Gd^3+^ and 1.45 for Eu^3+^/Sm^3+^ proved the high potential of Eu-IIMs for selective separation of Eu^3+^. Figure 5c shows the schematic separation of Eu^3+^ from other REEs. For extraction of Eu^3+^ from the membrane after adsorption, 1 M HCl solution was used.

Cui et al. [83] have recently reported the Gd^3+^ separation from a Gd^3+^-imprinted membrane (GIM) that was constructed with interlaced stacking of Gd^3+^-imprinted one-dimensional carbon nanotubes (GICNTs) and two-dimensional modified polydopamine-graphene oxide (PDA@GO). Polydopamine was added to graphene oxide to increase the interfacial adhesion. Next, the ion-imprinted polymerization was carried out on carbon nanotubes by a direct cross-link of linear polymers. Selectivity towards Gd (III) was obtained by passing a feed solution with a concentration of 60 mg/L Gd^3+^, 250 mg/L La^3+^ and 10 mg/L Eu^3+^. Isotherm and kinetic adsorption and time of permeation were investigated. The results show that the GICNT membrane has high permeation selectivity (2.91 La/Gd and 2.49 Eu/Gd) towards Gd^3+^. To prove the membrane performance, they measured the adsorption capacity of Gd^3+^ by passing the feed from the Gd^3+^-imprinted membrane (GIM) to the none-imprinted membrane (NIM). The results demonstrated the GIM had an adsorption capacity near 40 mg/g, while the NIM adsorption capacity was less, at under 15 mg/g. So, the Gd^3+^-imprinted membrane had a high potential capacity for Gd^3+^ separation. Figure 5d shows the scheme of Gd^3+^ separation from La^3+^ and Eu^3+^.

Though IIMs have many benefits for the extraction of REEs, such as the easy formation of porous structure and installation, low price and high flexibility, and their application for extraction of REEs seem promising in the future, their utilization is still scarce. Traditional ionic imprinted polymer membranes are suffering from low sites accessibility. Thus, template ions must be completely imprinted onto the membrane surfaces to achieve better imprinting efficiency [84,85]. Recently, a new three-dimensional (3D) wood-based Nd^3+^-imprinted with multilevel structure was introduced by Wu et al. [86]. Hereon, as illustrated in Figure 4a, the polydopamine (PDA)-modified layers were initially synthesized on the surfaces of basswood with a high macroporous structure. After that, the sandwich-like structure of 3DW-IIMs was made by carrying out an Nd^3+^-imprinted polymerization process. So, uniform and abundantly dispersed Nd^3+^-recognition sites were achieved on the surface, which improved the rebinding capacity (120.87 mg/g) and the high selectivity of Nd^3+^. Besides, they proved that the PDA-modified layers positively affected the rebinding capacity promotion of 3DW-IIMs, which caused much more creation of Nd^3+^-imprinted sites due to the PDA-modified surfaces. It was demonstrated that the retarded permeation mechanism had played an influential role in the selective separation of Nd^3+^. Figure 4b schematically illustrates that the adsorption of Nd^3+^ ions occurred during separation processes initially. Then, the same Nd^3+^ ions were bound onto the selective sites from the membrane. Inversely, non-template ions like Tb^3+^, Fe^3+^ and Dy^3+^ were transported through the membrane without any ion-imprinted resistance. Based on the obtained results, 3DW-IIMs showed excellent potential for the separation of REE ions. Table 3. summarizes the conditional operation of some IIMs for the separation of various REEs.

IIMs are very new methods for extraction of REEs. So, their application in industry is still not popular. However, they can be an interesting alternative of liquid membranes due to their high potential for separation of specific REE ions with high purified solution. Unfortunately, their production cost is high and new materials with low cost and easier synthesis methods need to be developed for IIMs production.

### 4.2. Nanocomposite Membranes

Recently, research into metal separation/extraction has led to a new class of materials called nanocomposites [88]. For example, pure polymeric membranes’ performance, selectivity, stability and permeability can be improved by incorporating nanoparticles such as iron, silver, aluminum, Palladium silica, zirconium, titanium and magnesium [89,90].

Also, preparation of polymeric nanocomposite membranes, applying nanomaterials based on carbon-containing material such as single-walled nanotubes (SWCNTs), multi-walled carbon nanotubes (MWCNTs) and graphene oxide (GO) nanoparticles, have been reported due to their enhanced and more effective performance [91,92,93].

Recently, Zolfonoun et al. [52] investigated the utilization of multi-walled carbon nanotubes membrane coated with cellulose acetate to extract REEs such as Y, Ce, La, Nd, Gd, Dy and Sm. The maximum capacity for Y^3+^, Ce^3+^, La^3+^, Nd^3+^, Dy^3+,^ Sm^3+^, Gd^3+^ and sorption by membrane was reported 33, 23, 28, 35, 31, 41 and 37 mg/g, respectively. The multi-walled (MWCNTs) nanotubes, because of high surface area in the range of 150 to 1500 m^2^/g, can easily adsorb REEs and facilitate the separation process. Referred to reported results, this method is a quick and simple approach and can be noticed as a suitable method for the determining and preconcentration of REEs.

**Figure 6:**
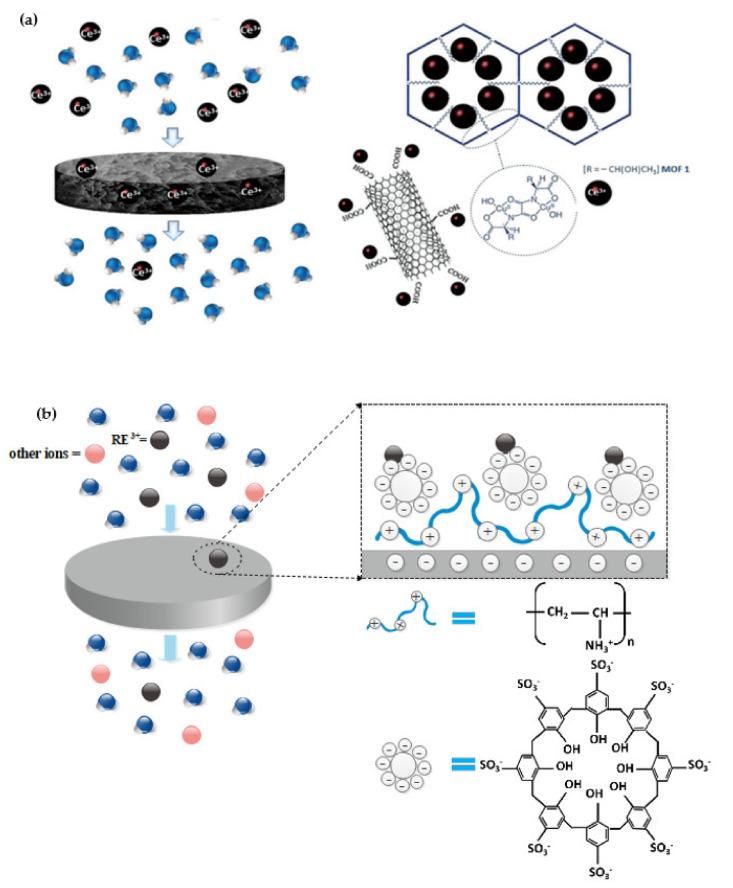
(**a**) Schematic of the recovery process of Ce^3+^ by BioMOF@SWCNT-BP membrane and purify of water stream, adapted from Ref. [94]. Copyright 2021 Wiley-VCH GmbH. and (**b**) The separation of REEs by a layer-by-layer composite of polyvinyl amine and p-octasulfonato-calix[8]arene. The polyvinyl amine with a positive charge is interacted with a negative surface charge and forms the first layer. In the following, the second layer is constructed by p-octasulfonato-calix[8]arene with a positive charge on the first layer. So, REE positive ions can be adsorbed on the surface of porous polymer membrane and other ions pass within the membrane, then extraction and purification are carried out [95].

Armentano et al. [94] also use a modern bio-MOF-based single-walled carbon nanotube buckypaper (SWCNTBP) to recover lanthanides. This MOF-carbon-based membrane introduced for the recovery of REEs for the first time exhibits high efficiency and performance in recovering Ce^3+^ from aqueous streams. Affected parameters such as concentration and PH were investigated. Results showed a sorption capacity of 263.30 mg/g for Ce^3+^ by BioMOF@SWCNT-BP membrane. As shown in Figure 6a, the separation mechanism of Ce^3+^ is involved in Ce^3+^ interaction with the COOH functional groups from SWCNTBP, followed by an uptake of MOF. As a result, BioMOF@SWCNT-BPs can be considered a cheap and effective membrane technique for minimizing pollution from streams and recovering REEs from water.

Additionally, due to its high accessibility and selectivity, a new type of layer-by-layer composite can be used to separate various kinds of REEs. Toutianoush et al. [95] have constructed a porous polymer membrane with layer-by-layer composition p-octasulfonato-calix[8]arene and polyvinyl amine on the surface of the membrane for separation of REEs, such as LaCl_3_ and YCl_3_. The schematic of membrane layers can be seen in Figure 6b. They exhibited that at suitable conditions. Permeation rates of YCl_3_, LaCl_3_, CeCl_3_, PrCl_3_ and SmCl_3_ were 0.08 × 10^−4^ m/s, 0.09 × 10^−4^ m/s, 0.101 × 10^−4^ m/s, 0.091 × 10^−4^ m/s and 0.093 × 10^−4^ m/s, respectively, which are extremely low. This can be ascribed to the complex formation of REE ions on the calixarenes sulfonate groups. So, the membrane is permeable for other ions but approximately impermeable for REEs ions. The strong rejection of the REE ions made the membrane useful for the enrichment of REEs ions.

As mentioned above, nanocomposite materials can modify membranes’ selectivity and recovery performance, but their application in the recovery of REEs from wastewater requires more academic research.

## 5. Rejection Mechanism

In a semipermeable membrane, separation of REEs in waste streams occurs by a rejection mechanism. Accordingly, when the stream is passing through the membrane, due to sieving mechanism (the difference between pore size of the membrane and REEs ions or REEs-complex size) or electrostatic rejection mechanism as a result of Donnan phenomenon and Nernst–Planck equation, the REEs that have been rejected from the surface of the membrane and the stream are purified. Then, the rejected REEs in a concentrated solution are extracted. These membranes have different characteristics, which are classified into nanofiltration (NF) with a pore size lower than 2 nm, ultrafiltration (UF) with a pore size between 2 nm and 50 nm and microfiltration (MF) with a pore size higher than 50 nm. Due to the large size of their pores, the MF membranes cannot reject REE ions, but they are usually used in a pre-treatment process to remove unfavorable ions such as sodium ions, which have a severe effect on the rejection of REEs from waste streams or acidic leachates.

A selective complexation method can be used to increase the REE ions rejection from these membranes for the information of complex between desired ions and water-soluble chelates. Therefore, the complexes are rejected quickly from the membrane surface, while the non-complexed ions transport through the membrane.

**Figure 7:**
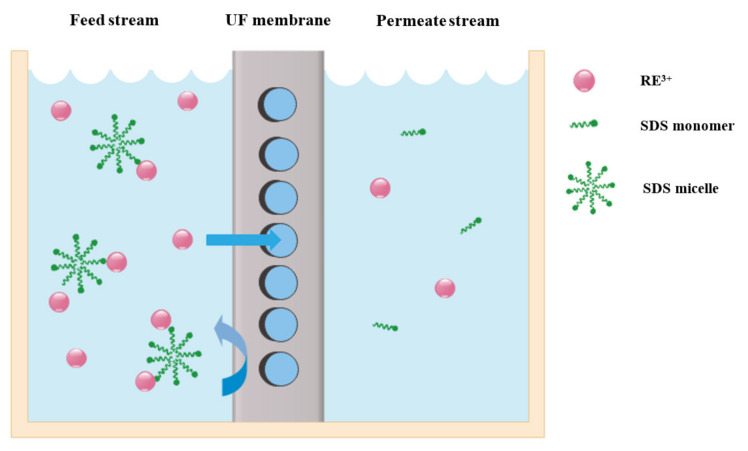
Schematic showing the rejection of REE ions from the surface of the membrane. The REE ions that formed complexes with SDS have a larger size than membrane pores, then they are rejected from the surface of the membrane, but free REE ions could easily pass through the membrane due to their smaller size [96].

Kose-Mutlu [96] reported the micelle enhanced ultrafiltration (MEUF) method for REE ions separation from a feed solution of mixed-metal using a sodium dodecyl sulfate (SDS) achelating agent. MEUF is a separation method that combines the solubilization and ultrafiltration process in which the desired ions are absorbed with micelles, so the large size ion-complexes reject from the surface of the UF membrane from the aqueous solution. Firstly, pre-treatment by pH adjustment with NaOH was carried out by microfiltration (MF). Then, two different membrane models, UP020 and UP150 with MWCO of 20 kDa and 150 kDa, respectively, were used to extract Tb^3+^, Nd^3+^, Eu^3+^, Er^3+^, Y^3+^ and Dy^3+^. Figure 7 shows the removal of REE ions from the surface of the membrane. They evaluated the SDS concentration as an important parameter on the MEUF at concentrations lower and higher than the CMC. The increase of the SDS concentration near CMC led to an increase of REE rejections and, at lower concentrations from CMC, the formation of micelles does not occur in the bulk suspension but near membrane surface due to the increase of concentrations. Concentration polarization took place and rejection of REEs was increased.

On the other hand, during the UF process, rejected molecules of the surfactant stick on the membrane surface and form a micelle aggregation layer (MAL) or a gel layer on the membrane surface and cause the REEs to bind on the surface. At higher SDS concentrations from CMC, the rejection of REE ions decreases, which is attributed to the shape change of micelles into worm-shaped with a smaller size that can easily pass through the membrane. UP020 membrane with an optimal parameter such as pH = 3.5, 60 min contact time at 25 °C, 3 bar operation pressure and SDS concentration of 8.0 mM, have a high REEs rejection of 97%.

Favre-Reguillon [97] et al., studied the Gd^3+^ rejection by NF-assisted complexation process using Desal G10 membrane and DTPA chelating agent. Without using DTPA, the rejection of Gd^3+^ was lower than 10%, but using DTPA in PH range of 1–3, an optimum condition (P = 4 bar, Gd^3+^ and DTPA concentration = 0.3 mM and T = 20), the rejection varied from 5% to 95%. This wide range of rejection was due to the formation and increased concentration of [Gd–DTPA]^2−^ complex as a function of PH.

Murthy and Choudhary [98] also studied the Rejection of Nd^3+^ using NF membrane (NF-300) with EDTA as complexation agent and SDS as surfactant. Using SDS and EDTA in feed solution led to an increase of Nd^3+^ rejection from 86.74 to 99.5% and 99.4%, respectively.

Polymer complexation UF process (PCUF) is another UF-assisted method for the rejection of metal ions that formed a large size of polymer complex. Various kinds of water-soluble polymers such as polyacrylic acid (PAA) or sodium salts of PAA can be used in this method. Gong et al. [99] used Amicon Ultra-15 centrifugal membrane with MWCO of 30 KDa and PAA as a polymer complexation agent to recover several REEs. They reported that more than 90% of REEs at PH between 8–9 by 30 mg/L of PAA were rejected from the membrane.

ValentinaInnocenzi et al. [100] used Micellar-enhanced monotubular ceramic membranes with sodium dodecyl sulfate (SDS) as an anionic surfactant for Y^3+^ and Zn^2+^ removal from from effluents obtained from waste electrical equipment recycling plants. Two model monotubular ceramic ultrafiltration membranes with MWCO of 1 kDa and 210 kDa were used. The concentration of Y^3+^ and Zn^2+^ in synthetic liquid was 30 mg/L and 2 mg/L, respectively. An excellent removal performance near 99% was obtained for both Y^3+^ and Zn^2+,^ which shows that retentate solution was rich in targeted ions that, for their recovery, further processed should be carried out.

Ceramic membranes with multi-layered structures as an inorganic membrane were also studied for the separation of REEs. J.López et al. [101] applied a TiO_2_ supported Al_2_O_3_ ceramic membrane with the specification of 44.92 cm^2^ active area in tubular configuration with 2 mm thickness and 6.5 mm internal diameter and 1 nm mean pore size. TiO2 was the active layer, and MWCO of the membrane was 200 Da with 4.5 and 5.5 isoelectric points (IEP). The membrane rejection mechanism can be attributed to the protonation and deprotonation of the TiO_2_ active layer surface groups (R–TiOH). The surface charge depends on pH, which is an acidic condition, the positively charged (R–TiOH^2+^) and negatively charged (R–TiO^−^) formed at basic condition. Rejection efficiencies of TiO_2_ ceramic membrane were approximately 60% which shows low selectivity of ceramic membrane for REEs because of the rejection of Al, Zn and Cu, alongside REE ions. A smaller pore size for ceramic membranes should be developed to reduce transportation of undesired ions, which helps increase REE ions rejections. As mentioned, ceramic membranes have attracted attention due to their superior chemical, thermal and mechanical resistance, hydrophilic nature, ease of cleaning, lower fouling and high operational lifetime, especially in harsh feedstock [102] that can be a good selection for REEs separation. However, due to the high cost of ceramic membranes, they are less used even in laboratory research.

In general, NF and UF membranes would be excellent for the treatment of very acidic streams, which are essential concerns for the environment due to the separation of monovalent ions such as H^+^, HSO_4_^−^, Na^+^, NO_3_^−^ and Cl^−^ that can pass freely through the membrane, alongside high rejection of REE trivalent ions at very low concentrations. Table 4 summarizes the information of some NF and UF membrane for REEs removal.

## 6. Conclusions

As the demand for rare earth elements (REEs) is intensively increasing worldwide, newly developed methods for extracting and recovering these precious metals seem considerably necessary. In this review, we investigated the efficient extraction of REEs by membrane process and their extraction mechanism. The various kinds of membrane processes, their feasibility, selectivity and extraction performance were also studied. The most commonly used methods for REEs are precipitation, ion exchange, adsorption, solvent extraction and membrane process. However, the precipitation, ion exchange and adsorption due to utilization of high chemical reagents are not economical. Moreover, they are not suitable for high concentration streams, so the amount of extractable REEs is low. The post treatment is also required to achieve high purified REEs. Currently, the solvent extraction method is the most common existing method for separating and extracting REEs. However, some restrictions such as low separation selectivity, low purity of the extracted elements and high energy consumption limits their application in industry and no drastic progress in scale-up applications has been made until now. Moreover, the remaining large amount of acidic and alkaline wastewater is a matter of concern. Therefore, researchers have recently focused on the extraction of REEs towards using a new and environmentally friendly method called membrane separation technology (MST). This method has excellent potential for scale-up extraction of REEs due to the use of lower chemical materials and easy operation. So, MST is more economical than other mentioned methods. Additionally, high concentrated streams with various conditions such as streams with different PH or with the presence of different undesirable ions, can be treated by this method.

Porous supported membranes that carrier embedded into its pores as the supported liquid membrane (SLM) is the most popular technique for the extraction of REEs. SLMs usually consume lower carrier and is more economical. Moreover, REEs’ extraction and stripping process coincide in one apparatus, which simplifies the extraction process, making it easier to use. However, the concern of carrier loss during extraction remains. On the other side, the selectivity of SLMs towards various REEs existed in a specific solution is low. So, using new extractants like ionic liquids, which have high selectivity and low toxicity than organic carriers, or other new strategies such as using two or three extractant simultaneously, could be the focus of future research within REEs extraction. Other membrane types called solid membranes are novel membranes for the extraction of REEs due to their easy preparation and green performance, which can be an effective alternative for liquid membranes. Polymer inclusion membranes (PIMs) are newly used for the extraction of various REEs. The extraction mechanism by these membranes is the permeation of REE ions through the membrane organic phase exactly like liquid membranes but the efficiency and performance period time are considerably higher due to immobilization of extractant in the membrane matrix. The future perspective of PIMs for REE extraction seems to be promising. However, more research on their feasibility and stability needs to be carried out for scale-up applications. For example, using new materials or synthesis processes can be the main topics for future research of PIMs.

Other types of new solid membranes are ion-imprinted membranes and nanocomposite membranes like carbon-based membranes or metal-organic framework (MOF) membranes; rare research has been carried out on their performance for recovery of REEs. However, they can be easily prepared, and because of their separation mechanism as facilitated/retarded permeation mechanism and adsorption of target ion in active sites within the membrane, they can be used for separation and extraction of a specific REE. The utilization of IIMs and MOF membranes for REE extractant is extremely new so it is necessary to conduct more research on their feasibility and performance. The main problem of IIMs is low stability of the template holes, especially for scale-up applications. The most important issue for the use of these membranes on an industrial scale is the high cost of manufacturing them that need to be considered. So new synthesis method needs to be developed for producing IIMs with more stable cavities and holes with economical price. Moreover, MOF and nanocomposite membranes have a more promising perspective because they had effective performance for water treatment in previous studies.

Other membranes, which typically act as a filter, separate the REE ions by rejection mechanism because of their pore size or surface charge of the membrane. These membranes are more useful for acidic solutions or leachate such as acid mine waters (AMWs), which have a low concentration of REEs and high concentration of hydrogen ions. These membranes usually allow the hydrogen ions to pass and reject the REE ions, so concentrated retentate of REE ions is obtained. Although NF and UF membranes are green approach for REEs recovery, they have low stability in aggressive acidic environments. Consequently, new materials like ceramic or nanocomposite materials can have positive effects on their development for use in industrial applications. NF membranes are more applied and UF membranes are usually integrated with other methods to improve the extraction of REEs. However, they currently have low efficiency for REEs recovery. As it turns out, the combination of these membrane with other new routes can be more promising. Table 5 summarizes the characteristics of various membranes examined in this review.

We believe that improvement and development on the performance of membranes due to their high potential properties for recovery of REEs will have a promising perspective in the future and can have positive effects on economic feasibility and the selectivity separation of REEs.

## Figures and Tables

**Table 1:** The compound of some supported liquid membranes for extraction and recovery of REEs.

Membrane Matrix	Carrier	Solvent	RE^3+^	Description	References
-	D2EHPA ^2^	-	Y^3+^ Nd^3+^ Dy^3+^	Development of a mathematical transient model and utilization of a new extraction selectivity definition to study the extraction and separation of REE ion mixtures with the FSHSLM process	[49]
PTFE ^1^	Aliquat-336	-	Gd^3+^ Nd^3+^	Improvement of 28.3% and 49.5% permeability coefficient for Gd^3+^ and Nd^3+^ extraction using hydrophobic nanoparticle SiO_2_	[65]
PVDF	TODGA ^3^	n-Octane	La^3+^ Ce^3+^ Pr^3+^ Nd^3+^	Very fast permeation and collection of more than 95.0% of REE ions using 0.1 M TODGA as carrier	[62]
polyvinylidene fluoride	D2EHPA	Kerosene	Nd^3+^	Development of a novel flat renewal supported liquid membrane (FRSLM) and obtaining an extraction percentage of 92.9 for Nd^3+^ in 75 min	[39]
Polypropylene (PP)	TODGA Cyanex 923 TBP ^4^	IsoparTM L	Nd^3+^ Pr^3+^ Dy^3+^	Use the SLM coupled with hollow supported fiber membrane for extraction of REE ions from permanent magnets wastes in the presence of non-REEs	[2]
-	[C_6_MIM][NTf_2_] TOPO ^5^ TPB	Kerosene	Nd^3+^ Pr^3+^	Achievement of the highest permeability coefficients with the synergistic extractant containing [C_6_MIM][NTf_2_] ionic liquid, TOPO and TPB carriers.	[64]
PTFE	combination of ionic liquids TBP D2EHPA	-	Ce^3+^	Observation of maximum permeation coefficient of 30%, 20% and 10% *v*/*v* for D2EHPA, TBP and C_6_MIM.NTF_2_ extractant	[66]
PVDF	DODGAA ^6^	[C_8_mim][Tf_2_N]	Nd^3+^ Dy^3+^	More than 99% extraction and purification of REEs from Fe ions with [C_8_mim][Tf2N] containing 10 mM DODGAA	[63]

^1^ polytetrafluoroethylene; ^2^ bis (2-ethylhexyl) hydrogen phosphate; ^3^ N,N,N′,N′-tetraoctyl diglycol amide; ^4^ Tributyl-phosphate; ^5^ trioctyl-phosphine-oxide; ^6^ N,N-dioctyldiglycol amic acid.

**Table 2:** The compound of some polymer inclusion membranes for extraction and recovery of REEs.

Membrane Matrix	Carrier	Plasticizer	RE^3+^	Description	References
CTA ^1^	D2EHPA and TBP ^4^	NPOE ^8^	La^3+^ Ce^3+^	Selectivity order (Ce/La) for TBP carrier = 1.4 Selectivity order (Ce/La) for D2EHPA carrier = 2.8	[75]
CTA	CMPO ^5^ and TODGA ^6^	-	Ce^3+^	Use of combined carriers with high effective transport of cerium ions	[76]
PVDF ^2^	P277	-	Lu^3+^	Selective separation of Lu^3+^ from La^3+^ and Sm^3+^ dependent on the PH of the feed solution	[7]
CTA	Noncyclic ionophores	-	Ce^3+^	A new combination of carriers for excellent separation of cerium ions	[77]
CTA	TODGA	NPOE	La^3+^ Eu^3+^ Lu^3+^	High transport percentage of La^3+^ = 60.4%, Eu^3+^ = 91.2% and Lu^3+^ = 98.0%	[71]
PVC ^3^	D2EHPA	-	La^3+^ Yb^3+^ Gd^3+^	Selective extraction of Yb^3+^ with thermodynamic extraction constants of Yb^3+^ = 92,700, Gd^3+^ = 85.5 and La^3+^ = 0.896 dependent on PH of the feed solution	[53]
PVDF	[A336][P507]	-	Lu^3+^	Use of bifunctional ionic liquid with good permeation coefficient of 2.80 µm/s compared to liquid-liquid extraction	[72]
EVOH ^7^	Cyanex272	-	Yb^3+^ Lu^3+^	High permeability coefficients of Lu^3+^ = 114.82 μm/s and Yb^3+^ = 156 μm/s	[69]

^1^ Cellulose-triacetate; ^2^ poly-vinylidene fluoride; ^3^ poly-vinyl chloride; ^4^ tributyl-phosphate-thylphosphine oxide; ^5^ octyl(phenyl)-N,N-diisobutylcarbamoylme; ^6^ N,N,N,N-tetraoctyl-3-oxapentanediamide; ^7^ polyvinyl-alcohol-co-ethylene; ^8^ 2-nitrophenyl-octyl-ether.

**Table 3:** Conditional operation of some ion-imprinted membranes for extraction and recovery of REEs.

Target Ion	Membrane Substance	PH of Adsorption	Initial Concentration (ppm)	Adsorption Capacity (mg/g)	Competitive Ions	References
Dy^3+^	chitosan membrane	7	50	23.3	Nd^3+^ Pr^3+^ Tb^3+^ Fe^3+^	[81]
Eu^3+^	Grapheme oxide (GO)/modified silicon dioxide (kSiO_2_) on PDA-modified substrate	7	50	101.14	La^3+^ Gd^3+^ Sm^3+^	[82]
Gd^3+^	Polydopamine/modified graphene oxide (PDA@GO) and carbon nanotubes (GICNTs) on cellulose membrane	7	60	-	La^3+^ Eu^3+^	[83]
Nd^3+^	polydopamine (PDA)-modified on basswood surfaces	7	90	120.87	Tb^3+^ Fe^3+^ Dy^3+^	[86]
Lu^3+^	4-vinylpyridine–acetylacetone/EDMA	5.5	20	64.2	Fe^3+^ −Mg^2+^ Ca^2+^ −Al^3+^ La^3+^ −Nd^3+^ Y^3+^ −Gd^3+^ Dy^3+^ −Tm^3+^ Lu^3+^	[87]

**Table 4:** A compound of some NF and UF membranes for removal and recovery of REEs.

Membrane Type	Active Layer	Organic Chelate	RE^3+^	MWCO (Da)	PH	Rejection (%)	References
NF270	Polyamide on a thin flm composite	-	La^3+^ Pr^3+^ Nd^3+^ Sm^3+^ Dy^3+^ Yb^3+^	180 ± 20	1	>98	[103]
Amicon Ultra-15 centrifugal filter units	-	PAA	La^3+^ Sm^3+^	30	7.5	>89	[99]
NF-300	Polyamide	EDTA SDS	Ce^3+^ Nd^3+^	300	2–10	>90	[98]
Single tube MembraloxO Tl-70 ceramic membrane	-	SDS	Y^3+^	1	5–6	99	[100]
UP020	-	SDS	Tb^3+^ Nd^3+^ Eu^3+^ Er^3+^ Y^3+^ Dy^3+^	20	3.5	>97	[96]
TiO_2_ supported Al_2_O_3_ ceramic membrane	TiO_2_	-	REE ions Al^3+^ Zn^2+^ Cu^2+^	200	-	60	[101]

**Table 5:** The characteristics of various membranes for REEs extraction.

Membrane Type	Functional PH	Selectivity	Life Time	Price
Supported liquid membrane (SLM)	Acidic and neutral conditions	Higher for LREEs	Low due to pore blockage	Low for continuous extraction process, few consuption chemical materials and easily operation
Polymer inclusion membrane (PIM)	Acidic and neutral conditions	Higher for HREEs	Low due to fouling, low thermal, and chemical stability	Low for continuous extraction process, few consuption chemical materials and easily operation
Ion imprinted membrane (IIM)	5–7	High	Low due to loss of active sites after a period	High for production cost and needed post treatment
Nanocomposite membrane	Acidic and neutral conditions	low	Long for high chemical and thermal stability	High for production cost
Ultrafiltration	More suitable for high acidic conditions	Low	Long for high chemical and mechanical stability	High for production cost and needed pre-treatment
Nanofiltration	More suitable for high acidic conditions	Low	Long for high chemical and mechanical stability	High for production cost, needed pre-treatment and consumption of chelating agent

## Data Availability

The study did not report any data.

## References

[1] Pramanik B.K., Nghiem L.D., Hai F.I. (2020). Extraction of strategically important elements from brines: Constraints and opportunities. Water Res..

[2] Kim D., Powell L., Delmau L.H., Peterson E.S., Herchenroeder J., Bhave R.R. (2016). A supported liquid membrane system for the selective recovery of rare earth elements from neodymium-based permanent magnets. Sep. Sci. Technol..

[3] Ali A.H., Dakroury G.A., Hagag M.S., Abdo S.M., Allan K.F. (2021). Sorption of Some Rare Earth Elements from Acidic Solution onto Poly (acrylic acid–co-acrylamide/16, 16-dimethylheptadecan-1-amine) Composite. J. Polym. Environ..

[4] Chen L., Wu Y., Dong H., Meng M., Li C., Yan Y., Chen J. (2018). An overview on membrane strategies for rare earths extraction and separation. Sep. Purif. Technol..

[5] Li F., Yang Z., Weng H., Chen G., Lin M., Zhao C. (2018). High efficient separation of U(VI) and Th(IV) from rare earth elements in strong acidic solution by selective sorption on phenanthroline diamide functionalized graphene oxide. Chem. Eng. J..

[6] Balaram V. (2019). Rare earth elements: A review of applications, occurrence, exploration, analysis, recycling, and environmental impact. Geosci. Front..

[7] Huang S., Chen J., Zou D. (2021). A preliminary study of polymer inclusion membrane for lutetium(III) separation and membrane regeneration. J. Rare Earths.

[8] Liu J., Martin P.F., Peter McGrail B. (2021). Rare-earth element extraction from geothermal brine using magnetic core-shell nanoparticles-techno-economic analysis. Geothermics.

[9] Hidayah N.N., Abidin S.Z. (2017). The evolution of mineral processing in extraction of rare earth elements using solid-liquid extraction over liquid-liquid extraction: A review. Miner. Eng..

[10] Elbashier E., Mussa A., Hafiz M., Hawari A.H. (2021). Recovery of rare earth elements from waste streams using membrane processes: An overview. Hydrometallurgy.

[11] Asadollahzadeh M., Torkaman R., Torab-Mostaedi M. (2020). Extraction and Separation of Rare Earth Elements by Adsorption Approaches: Current Status and Future Trends. Sep. Purif. Rev..

[12] Zhao Z., Qiu Z., Yang J., Lu S., Cao L., Zhang W., Xu Y. (2017). Recovery of rare earth elements from spent fluid catalytic cracking catalysts using leaching and solvent extraction techniques. Hydrometallurgy.

[13] Kose Mutlu B., Cantoni B., Turolla A., Antonelli M., Hsu-Kim H., Wiesner M.R. (2018). Application of nanofiltration for Rare Earth Elements recovery from coal fly ash leachate: Performance and cost evaluation. Chem. Eng. J..

[14] Drobniak A., Mastalerz M. (2022). Rare Earth Elements: A brief overview. Indiana J. Earth.

[15] Hammache Z., Bensaadi S., Berbar Y., Audebrand N., Szymczyk A., Amara M. (2021). Recovery of rare earth elements from electronic waste by diffusion dialysis. Sep. Purif. Technol..

[16] Pavón S., Fortuny A., Coll M.T., Sastre A.M. (2019). Improved rare earth elements recovery from fluorescent lamp wastes applying supported liquid membranes to the leaching solutions. Sep. Purif. Technol..

[17] Ni’am A.C., Wang Y.-F., Chen S.-W., Chang G.-M., You S.-J. (2020). Simultaneous recovery of rare earth elements from waste permanent magnets (WPMs) leach liquor by solvent extraction and hollow fiber supported liquid membrane. Chem. Eng. Process.—Process Intensif..

[18] Dong Z., Mattocks J.A., Deblonde G.J.P., Hu D., Jiao Y., Cotruvo J.A., Park D.M. (2021). Bridging Hydrometallurgy and Biochemistry: A Protein-Based Process for Recovery and Separation of Rare Earth Elements. ACS Cent. Sci..

[19] Goode J.R. Options for the separation of rare earth elements. Proceedings of the IMPC 2016 28th International Mineral Processing Congress.

[20] Hassas B.V., Rezaee M., Pisupati S.V. (2021). Effect of various ligands on the selective precipitation of critical and rare earth elements from acid mine drainage. Chemosphere.

[21] Park D., Middleton A., Smith R., Deblonde G., Laudal D., Theaker N., Hsu-Kim H., Jiao Y. (2020). A biosorption-based approach for selective extraction of rare earth elements from coal byproducts. Sep. Purif. Technol..

[22] Smith R.C., Taggart R.K., Hower J.C., Wiesner M.R., Hsu-Kim H. (2019). Selective Recovery of Rare Earth Elements from Coal Fly Ash Leachates Using Liquid Membrane Processes. Environ. Sci. Technol..

[23] Pramanik B.K., Shu L., Jegatheesan J., Shah K., Haque N., Bhuiyan M.A. (2019). Rejection of rare earth elements from a simulated acid mine drainage using forward osmosis: The role of membrane orientation, solution pH, and temperature variation. Process Saf. Environ. Prot..

[24] Oleinikova M., Muñoz M. (2000). Transport of rare earth metal ions through activated composite membranes containing DEHPA. Solvent Extr. Ion Exch..

[25] Tian M., Jia Q., Liao W. (2013). Studies on Synergistic solvent extraction of rare earth elements from nitrate medium by mixtures of 8-hydroxyquinoline with Cyanex 301 or Cyanex 302. J. Rare Earths.

[26] Zhou H., Wang Y., Guo X., Dong Y., Su X., Sun X. (2018). The recovery of rare earth by a novel extraction and precipitation strategy using functional ionic liquids. J. Mol. Liq..

[27] Kegl T., Košak A., Lobnik A., Novak Z., Kralj A.K., Ban I. (2020). Adsorption of rare earth metals from wastewater by nanomaterials: A review. J. Hazard. Mater..

[28] Korenevsky A.A., Sorokin V.V., Karavaiko G.I. (1999). Biosorption of rare earth elements. Process Metall..

[29] Xin W., Lin C., Fu L., Kong X.Y., Yang L., Qian Y., Zhu C., Zhang Q., Jiang L., Wen L. (2021). Nacre-like Mechanically Robust Heterojunction for Lithium-Ion Extraction. Matter.

[30] Brewer A., Chang E., Park D.M., Kou T., Li Y., Lammers L.N., Jiao Y. (2019). Recovery of Rare Earth Elements from Geothermal Fluids through Bacterial Cell Surface Adsorption. Environ. Sci. Technol..

[31] Li C., Ramasamy D.L., Sillanpää M., Repo E. (2021). Separation and concentration of rare earth elements from wastewater using electrodialysis technology. Sep. Purif. Technol..

[32] Xie Z., Chen Q., Zhao L. (2013). Extraction of trace rare earth by liquid surfactant membranes from phosphate rock. Zhongguo Xitu Xuebao/J. Chin. Rare Earth Soc..

[33] Davoodi-Nasab P., Rahbar-Kelishami A., Safdari J., Abolghasemi H. (2017). Application of emulsion nanofluids membrane for the extraction of gadolinium using response surface methodology. J. Mol. Liq..

[34] Wang J., Dang Y., Fei D., Fan W., Dong W. (2012). Recovery of trace lanthanum from phosphoric acid using liquid membrane extraction. Zhongguo Xitu Xuebao/J. Chin. Rare Earth Soc..

[35] Tang J., Wai C.M. (1989). Transport of trivalent lanthanides through a surfactant membrane containing an ionizable macrocyclic polyether. J. Membr. Sci..

[36] Yang X.J., Fane A.G., Soldenhoff K. (2003). Comparison of liquid membrane processes for metal separations: Permeability, stability, and selectivity. Ind. Eng. Chem. Res..

[37] Gaikwad A.G., Rajput A.M. (2010). Transport of yttrium metal ions through fibers supported liquid membrane solvent extraction. J. Rare Earths.

[38] Pei L., Yao B., Zhang C. (2009). Transport of Tm(III) through dispersion supported liquid membrane containing PC-88A in kerosene as the carrier. Sep. Purif. Technol..

[39] Pei L., Wang L., Yu G. (2012). Study on a novel flat renewal supported liquid membrane with D2EHPA and hydrogen nitrate for neodymium extraction. J. Rare Earths.

[40] Pei L., Wang L., Yu G. (2011). Separation of Eu(III) with supported dispersion liquid membrane system containing D2EHPA as carrier and HNO_3_ solution as stripping solution. J. Rare Earths.

[41] Doležal J., Moreno C., Hrdlička A., Valiente M. (2000). Selective transport of lanthanides through supported liquid membranes containing non-selective extractant, di-(2-ethylhexyl)phosphoric acid, as a carrier. J. Membr. Sci..

[42] Pei L., Yao B., Fu X. (2009). Study on transport of Dy(III) by dispersion supported liquid membrane. J. Rare Earths.

[43] Ambare D.N., Ansari S.A., Anitha M., Kandwal P., Singh D.K., Singh H., Mohapatra P.K. (2013). Non-dispersive solvent extraction of neodymium using a hollow fiber contactor: Mass transfer and modeling studies. J. Membr. Sci..

[44] Ramakul P., Mooncluen U., Yanachawakul Y., Leepipatpiboon N. (2012). Mass transport modeling and analysis on the mutual separation of lanthanum(III) and cerium(IV) through a hollow fiber supported liquid membrane. J. Ind. Eng. Chem..

[45] Yadav K.K., Anitha M., Singh D.K., Kain V. (2018). NdFeB magnet recycling: Dysprosium recovery by non-dispersive solvent extraction employing hollow fibre membrane contactor. Sep. Purif. Technol..

[46] Pei L., Wang L., Guo W., Zhao N. (2011). Stripping dispersion hollow fiber liquid membrane containing PC-88A as carrier and HCl for transport behavior of trivalent dysprosium. J. Membr. Sci..

[47] Geist A., Weigl M., Müllich U., Gompper K. (2003). Application of novel extractants for actinide(III)/lanthanide(III) separation in hollow-fibre modules. Membr. Technol..

[48] Kubota F., Kakoi T., Goto M., Furusaki S., Nakashio F., Hano T. (2000). Permeation behavior of rare earth metals with a calix[4]arene carboxyl derivative in a hollow-fiber membrane. J. Membr. Sci..

[49] Martínez J., Rodríguez Varela R., Forsberg K., Rasmuson Å. (2018). Factors influencing separation selectivity of rare earth elements in flat sheet supported liquid membranes. Chem. Eng. Sci..

[50] Li X., Sun Y. (2005). Progress in solid-liquid extraction resin for separation of rare earth elements. J. Rare Earths.

[51] Nghiem L.D., Mornane P., Potter I.D., Perera J.M., Cattrall R.W., Kolev S.D. (2006). Extraction and transport of metal ions and small organic compounds using polymer inclusion membranes (PIMs). J. Membr. Sci..

[52] Zolfonoun E., Yousefi S.R. (2016). Simultaneous Determination of Rare Earth Elements by ICP OES After On-Line Enrichment Using Multi-Walled Carbon Nanotubes Coated Cellulose Acetate Membrane. J. Braz. Chem. Soc..

[53] Croft C.F., Almeida M.I.G.S., Cattrall R.W., Kolev S.D. (2018). Separation of lanthanum(III), gadolinium(III) and ytterbium(III) from sulfuric acid solutions by using a polymer inclusion membrane. J. Membr. Sci..

[54] Yusoff M.M., Mostapa N.R.N., Sarkar M.S., Biswas T.K., Rahman M.L., Arshad S.E., Sarjadi M.S., Kulkarni A.D. (2017). Synthesis of ion imprinted polymers for selective recognition and separation of rare earth metals. J. Rare Earths.

[55] Kala R., Biju V.M., Rao T.P. (2005). Synthesis, characterization, and analytical applications of erbium(III) ion imprinted polymer particles prepared via γ-irradiation with different functional and crosslinking monomers. Anal. Chim. Acta.

[56] Zhang N., Hu B., Huang C. (2007). A new ion-imprinted silica gel sorbent for on-line selective solid-phase extraction of dysprosium(III) with detection by inductively coupled plasma-atomic emission spectrometry. Anal. Chim. Acta.

[57] Wang J., Wei J., Li J. (2016). Straw-supported ion imprinted polymer sorbent prepared by surface imprinting technique combined with AGET ATRP for selective adsorption of La3+ ions. Chem. Eng. J..

[58] Hou H., Jing Y., Wang Y., Wang Y., Xu J., Chen J. (2015). Solvent extraction performance of Ce(III) from chloride acidic solution with 2-ethylhexyl phosphoric acid-2-ethylhexyl ester (EHEHPA) by using membrane dispersion micro-extractor. J. Rare Earths.

[59] Hou H., Wang Y., Xu J., Chen J. (2013). Solvent extraction of La(III) with 2-ethylhexyl phosphoric acid-2-ethylhexyl ester (EHEHPA) by membrane dispersion micro-extractor. J. Rare Earths.

[60] Qiu S., Xue M., Zhu G. (2014). Metal–organic framework membranes: From synthesis to separation application. Chem. Soc. Rev..

[61] Carboni M., Abney C.W., Liu S., Lin W. (2013). Highly porous and stable metal–organic frameworks for uranium extraction. Chem. Sci..

[62] Xu D., Shah Z., Sun G., Peng X., Cui Y. (2019). Recovery of rare earths from phosphate ores through supported liquid membrane using N, N, N′, N′-tetraoctyl diglycol amid. Miner. Eng..

[63] Baba Y., Kubota F., Kamiya N., Goto M. (2011). Selective recovery of dysprosium and neodymium ions by a supported liquid membrane based on ionic liquids. Solvent Extr. Res. Dev..

[64] Asadollahzadeh M., Torkaman R., Torab-Mostaedi M., Hemmati A., Ghaemi A. (2020). Efficient recovery of neodymium and praseodymium from NdFeB magnet-leaching phase with and without ionic liquid as a carrier in the supported liquid membrane. Chem. Pap..

[65] Mohammad Tehrani B., Rahbar-Kelishami A. (2019). A novel mass transfer coefficient correlation for improved rare earth metals extraction via supported nanoliquids membrane. Chem. Eng. Process.–Process Intensif..

[66] Asadollahzadeh M., Torkaman R., Torab-Mostaedi M., Ghaemi A., Hemmati A. (2020). Green imidazolium ionic liquid selectively facilitates Ce(III) ion transport through supported liquid membrane. Int. J. Environ. Anal. Chem..

[67] Bhattacharyya A., Mohapatra P.K., Hassan P.A., Manchanda V.K. (2011). Studies on the selective Am3+ transport, irradiation stability and surface morphology of polymer inclusion membranes containing Cyanex-301 as carrier extractant. J. Hazard. Mater..

[68] Baczyńska M., Waszak M., Nowicki M., Przadka D., Borysiak S., Regel-Rosocka M. (2018). Characterization of Polymer Inclusion Membranes (PIMs) Containing Phosphonium Ionic Liquids as Zn(II) Carriers. Ind. Eng. Chem. Res..

[69] Huang S., Chen J., Chen L., Zou D., Liu C. (2020). A polymer inclusion membrane functionalized by di(2-ethylhexyl) phosphinic acid with hierarchically ordered porous structure for Lutetium(III) transport. J. Membr. Sci..

[70] Chen L., Dong H., Pan W., Dai J., Dai X., Pan J. (2021). Poly (vinyl alcohol-co-ethylene) (EVOH) modified polymer inclusion membrane in heavy rare earths separation with advanced hydrophilicity and separation property. Chem. Eng. J..

[71] Ansari S.A., Mohapatra P.K., Manchanda V.K. (2010). Cation transport across plasticized polymeric membranes containing N, N, N′, N′-tetraoctyl-3-oxapentanediamide (TODGA) as the carrier. Desalination.

[72] Chen L., Chen J. (2016). Asymmetric Membrane Containing Ionic Liquid [A336][P507] for the Preconcentration and Separation of Heavy Rare Earth Lutetium. ACS Sustain. Chem. Eng..

[73] Lozano L.J., Godínez C., Ríos A.P.d.l., Hernández-Fernández F.J., Sánchez-Segado S., Alguacil F.J. (2011). Recent advances in supported ionic liquid membrane technology. Membr. Sci..

[74] Liu T., Chen J. (2021). Extraction and separation of heavy rare earth elements: A review. Sep. Purif. Technol..

[75] Makowka A., Pospiech B. (2019). Synthesis of Polymer Inclusion Membranes based on Cellulose Triacetate for Recovery of Lanthanum(III) from Aqueous Solutions. Autex Res. J..

[76] Kusumocahyo S.P., Kanamori T., Sumaru K., Aomatsu S., Matsuyama H., Teramoto M., Shinbo T. (2004). Development of polymer inclusion membranes based on cellulose triacetate: Carrier-mediated transport of cerium(III). J. Membr. Sci..

[77] Hiratani K., Kusumocahyo S.P., Kameta N., Sugaya K., Shinbo T., Kanamori T. (2005). Synthesis of noncyclic carriers for cerium ion transport through polymer inclusion membrane. Chem. Lett..

[78] Guo W., Ngo H.H., Li J. (2012). A mini-review on membrane fouling. Bioresour. Technol..

[79] Kojiro S., Iori F., Kiyoshi F., Hiroyuki O., Tsuyoshi S., Tatsuya O., Yoshinari B., Hirochika N. (2016). Extraction behavior of Rare-earth elements using a Mono-alkylated Diglycolamic acid extractant. Solvent Extr. Res. Dev. Jpn..

[80] Abdollahi H., Maleki S., Sayahi H., Gharabaghi M., Darvanjooghi M.H.K., Magdouli S., Brar S.K. (2021). Superadsorbent Fe3O4-coated carbon black nanocomposite for separation of light rare earth elements from aqueous solution: GMDH-based Neural Network and sensitivity analysis. J. Hazard. Mater..

[81] Liu E., Xu X., Zheng X., Zhang F., Liu E., Li C. (2017). An ion imprinted macroporous chitosan membrane for efficiently selective adsorption of dysprosium. Sep. Purif. Technol..

[82] Lu J., Wu Y., Lin X., Gao J., Dong H., Chen L., Qin Y., Wang L., Yan Y. (2018). Anti-fouling and thermosensitive ion-imprinted nanocomposite membranes based on grapheme oxide and silicon dioxide for selectively separating europium ions. Hazard. Mater..

[83] Cui K., Gao B., Tai M., Su B. (2019). A facile bionic strategy towards Gd(III)-imprinted membranes via interlaced stacking of one-dimensional/two-dimensional nanocomposite materials. J. Taiwan Inst. Chem. Eng..

[84] Zheng X., Zhang Y., Zhang F., Li Z., Yan Y. (2018). Dual-template docking oriented ionic imprinted bilayer mesoporous films with efficient recovery of neodymium and dysprosium. J. Hazard. Mater..

[85] Wu Y., Lu J., Xing W., Ma F., Gao J., Lin X., Yu C., Yan M. (2020). Double-layer-based molecularly imprinted membranes for template-dependent recognition and separation: An imitated core-shell-based synergistic integration design. Chem. Eng. J..

[86] Wu Y., Lin R., Ma F., Xing W., Pan J. (2021). Three-dimensional macroporous wood-based selective separation membranes decorated with well-designed Nd(III)-imprinted domains: A high-efficiency recovery system for rare earth element. J. Colloid Interface Sci..

[87] Lai X., Hu Y., Fu Y., Wang L., Xiong J. (2012). Synthesis and Characterization of Lu(III) Ion Imprinted Polymer. Inorg. Organomet. Polym. Mater..

[88] Comandella D., Bonani W., Ciscar J.B., Ponti J., Cologna M., Popa K., Gilliland D. (2021). Recovery of rare earth elements by nanometric CeO2embedded into electrospun PVA nanofibres. RSC Adv..

[89] Ouda M., Hai A., Krishnamoorthy R., Govindan B., Othman I., Kui C.C., Choi M.Y., Hasan S.W., Banat F. (2022). Surface tuned polyethersulfone membrane using an iron oxide functionalized halloysite nanocomposite for enhanced humic acid removal. Environ. Res..

[90] Sarkar S., Chakraborty S. (2021). Nanocomposite polymeric membrane a new trend of water and wastewater treatment: A short review. Groundw. Sustain. Dev..

[91] Ng L.Y., Chua H.S., Ng C.Y. (2021). Incorporation of graphene oxide-based nanocomposite in the polymeric membrane for water and wastewater treatment: A review on recent development. J. Environ. Chem. Eng..

[92] Kim Y.-S., Seo K.-S., Choi S.-H. (2016). Polymeric nanocomposite proton exchange membranes prepared by radiation-induced polymerization for direct methanol fuel cell. Radiat. Phys. Chem..

[93] Liang B., He X., Hou J., Li L., Tang Z. (2019). Membrane Separation in Organic Liquid: Technologies, Achievements, and Opportunities. Adv. Mater..

[94] Tursi A., Mastropietro T.F., Bruno R., Baratta M., Ferrando-Soria J., Mashin A.I., Nicoletta F.P., Pardo E., De Filpo G., Armentano D. (2021). Synthesis and Enhanced Capture Properties of a New BioMOF@SWCNT-BP: Recovery of the Endangered Rare-Earth Elements from Aqueous Systems. Adv. Mater. Interfaces.

[95] Toutianoush A., El-Hashani A., Schnepf J., Tieke B. (2005). Multilayer membranes of p-sulfonato-calix[8]arene and polyvinylamine and their use for selective enrichment of rare earth metal ions. Appl. Surf. Sci..

[96] Kose-Mutlu B., Hsu-Kim H., Wiesner M.R. (2020). Separation of rare earth elements from mixed-metal feedstocks by micelle enhanced ultrafiltration with sodium dodecyl sulfate. Environ. Technol..

[97] Sorin A., Favre-Reguillon A., Pellet-Rostaing S., Sba M., Szymczyk A., Fievet P., Lemaire M. (2005). Rejection of Gd(III) by nanofiltration assisted by complexation on charged organic membrane: Influences of pH, pressure, flux, ionic strength and temperature. Membr. Sci..

[98] Murthy Z.V.P., Choudhary A. (2011). Application of nanofiltration to treat rare earth element (neodymium) containing water. J. Rare Earths.

[99] Duan H., Lin J., Gong Z., Huang J., Yang S. (2015). Removal of high-salinity matrices through polymer-complexation–ultrafiltration for the detection of trace levels of REEs using inductively coupled plasma mass spectrometry. Talanta.

[100] Innocenzi V., Prisciandaro M., Tortora F., Mazziotti di Celso G., Vegliò F. (2018). Treatment of WEEE industrial wastewaters: Removal of yttrium and zinc by means of micellar enhanced ultra filtration. Waste Manag..

[101] López J., Reig M., Vecino X., Gibert O., Cortina J.L. (2020). Comparison of acid-resistant ceramic and polymeric nanofiltration membranes for acid mine waters treatment. Chem. Eng. J..

[102] Ağtaş M., Dilaver M., Koyuncu İ. (2021). Ceramic membrane overview and applications in textile industry: A review. Water Sci. Technol..

[103] López J., Reig M., Gibert O., Cortina J.L. (2019). Recovery of sulphuric acid and added value metals (Zn, Cu and rare earths) from acidic mine waters using nanofiltration membranes. Sep. Purif. Technol..

